# Safety Assessment of Electronic Cigarettes and Their Relationship with Cardiovascular Disease

**DOI:** 10.3390/ijerph15010075

**Published:** 2018-01-05

**Authors:** Guangwei Zhang, Zhangli Wang, Kai Zhang, Rui Hou, Chunli Xing, Qi Yu, Enqi Liu

**Affiliations:** 1 Shaanxi Key Laboratory of Ischemic Cardiovascular Disease, Department of Public Health & College of Clinical Medicine, Xi’an Medical University, No.1 Xinwang Road, Xi’an 710021, China; zgw_1979@163.com (G.Z.); 18700423907@163.com (Z.W.); 18309283145@163.com (K.Z.); 18291457642@163.com (R.H.); 18291931494@163.com (C.X.); qiyu6028@hotmail.com (Q.Y.); 2 Laboratory Animal Center, Xi’an Jiaotong University Health Science Center, Xi’an Jiaotong University, Xi’an 710061, China

**Keywords:** electronic cigarettes, security, atherosclerosis, smoking cessation

## Abstract

Smoking leads to the occurrence and development of a variety of diseases. Most importantly, it is an independent risk factor of cardiovascular atherosclerosis. In recent years, electronic cigarettes have become a popular alternative to traditional cigarettes, since modern micro-electronic techniques provide the possibility of simulating the process of traditional smoking. Additionally, it is convenient and fashionable. Nevertheless, comments about the safety of electronic cigarettes remain controversial. Although the research about electronic cigarettes increased exponentially, there has been no systematic study of its safety. The aim of the current study is to review the literature reports about the safety of electronic cigarettes, and to understand their hazards and disadvantages. It was found that most of the current research about electronic cigarettes comprises short-term and in vitro studies. There are few reports of in vivo and long-term studies. Notably, the level of harmful components such as volatile organic compounds, tobacco-specific nitrosamines and heavy metals in electronic cigarettes are even higher than in traditional cigarettes. Therefore, the harm of electronic cigarettes should not be underestimated. In conclusion, the question of whether electronic cigarettes are a safe and sufficient substitute for traditional smoking needs further investigation.

## 1. Introduction

Electronic cigarette (E-cigarette) is a term that usually refers to a kind of electronic product that imitates traditional cigarettes in terms of their appearance, taste, aroma and other aspects. They are powered by batteries, conveying nicotine to the respiratory system via a heating or atomizing method, which leads to the same physiological and psychological feeling as traditional cigarettes [1]. In 2014, the World Health Organization (WHO) defined devices that could release nicotine (also known as E-cigarettes) as products that suck nicotine aerosol through a cigarette holder or other components [2].

**Figure 1:**
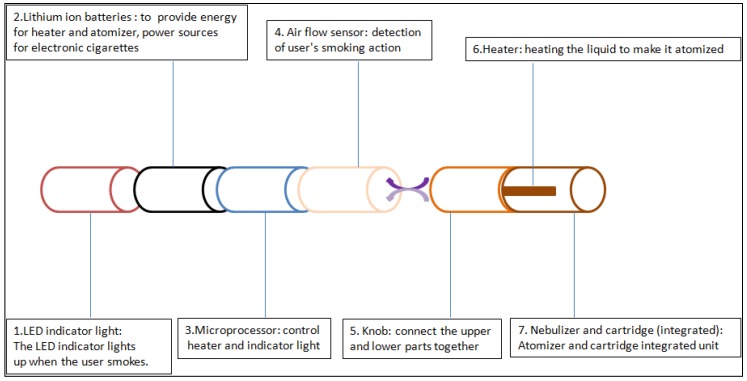
Diagram of electronic cigarette design.

Traditional tobacco products mainly release chemicals for people to smoke via chemical reaction triggered by burning; while E-cigarettes output nicotine and aromatic substances by means of electronic heating, and are electronic devices that enable smokers to have the same physiological feeling as smoking real cigarettes. They were first invented by Chinese pharmacist Han Li, and have gradually flowed into Europe and America. Although the exact structural design of E-cigarettes is constantly changing, the structure is composed of three parts: typically, a battery, atomizer, and liquid storage tank (cartridge) (as shown in Figure 1) [3]. When people smoke E-cigarettes, the electronic device in the E-cigarette smoke will atomize the e-liquid (the solution used to generate the aerosol) in the cartridge, and then the smoke will enter the body through the mouth. In recent years, many enterprises have entered the field of E-cigarettes. In order to cater to the diverse needs of people, E-cigarettes have been developed with a variety of tastes. The production technology of E-cigarettes has been constantly improved, and its market share continues to expand. 

The e-liquid of the E-cigarette is usually a liquid mixture of propylene glycol, glycerin, nicotine, essence and some other chemical substances. E-cigarette sellers have always packaged E-cigarettes as harmless smoking-cessation products or cigarette substitutes without harmful substances such as tar, carbon monoxide, suspended particles, etc. However, up to now, the research about the safety of E-cigarettes has been scarce, and there is no systematic review focusing on the safety assessment of E-cigarettes worldwide [4]. In particular, it is still unclear whether aerosols produced by electronic smoke do harm to our health. Furthermore, it’s controversial that E-cigarettes emit only harmless steam [5]. Although more and more research shows that the known dangers of electronic smoke are much smaller than for traditional cigarettes, it is uncertain how dangerous the potential toxic effects of E-cigarettes are.

Nicotine is highly involved in the development of cardiovascular disease, especially atherosclerosis. Although tar and other harmful ingredients have been removed in the e-liquid, more work should be done to evaluate its safety, due to the existence of nicotine and other potential hazards such as aerosol particles and different flavor additives in the e-liquid. These may increase the risk of cardiovascular disease. Moreover, the number and size distribution of particles from E-cigarettes are similar to those from traditional cigarettes, and some E-cigarettes produce more particles than traditional cigarettes. Particles may cause lung and systemic inflammation, and increase the risk of cardiovascular disease, respiratory disease and death [5].

As a result, it is of great importance to evaluate whether E-cigarettes can become a substitute for traditional cigarettes and become a safe and reliable way to quit smoking. Therefore, the aim of current study is to systematically review the literature reports about the safety of electronic cigarettes and to understand its hazards and disadvantages.

## 2. Research Content of E-Cigarettes

### 2.1. The Development of E-Cigarettes

With the extension of people’s health consciousness and the worldwide campaigns for a blanket smoking ban, E-cigarettes first appeared in the public arena. The basic concept of E-cigarettes is that they are smokeless, non-tobacco cigarettes, and this was proposed by Herbert Gilbert in 1963, and patented in 1965, but did not enter the market [6]. In 2003, a Chinese pharmacist Han Li applied for and obtained a patent for “a non-flammable electronic atomizing cigarette” [7]. In 2004, the Ruyan Company of China began to develop and sell E-cigarettes. E-cigarettes gradually became commercially available. In 2007, the international patent of E-cigarettes was obtained [8]. Since then, the E-cigarettes have continued to develop, spreading from China to Europe, America, Japan and other countries. By 2014, E-cigarettes were being sold in more than 60 countries and regions all over the world, and were being sought after by more and more consumers of different ages. China has become the main production place of E-cigarettes, and about 90% of E-cigarettes in the world are produced in Shenzhen [9]. In recent years, in response to the different demands of E-cigarette consumers, along with the broad market requirements, E-cigarettes have developed rapidly. Different electronic products have been put on the market, and the replacement cycle of similar products is very short. 

### 2.2. The Classification of E-Cigarettes in Different Countries Based on Its Properties

The regulation strategy for E-cigarettes is not unified all over the world. Except for some countries that either completely prohibit E-cigarettes or have not yet declared, the other countries variously regard it as tobacco, a pharmaceutical product, or as an ordinary consumer product (see Table 1) [10]. The following part will introduce the E-cigarette regulation strategy in different countries. Some countries adopt a “classification regulation” strategy, classifying the E-cigarettes according to the existence of nicotine or tobacco extract in e-liquid or, the content and concentration of nicotine. Different types of E-cigarettes comply with different control policies. The classification standards of E-cigarettes in different countries are quite different. Generally, E-cigarettes can be classified into one of three categories: tobacco, pharmaceutical products, or ordinary consumer goods.

#### 2.2.1. Tobacco Products

The United States, South Korea and Singapore regard E-cigarettes as tobacco under control (see Table 1). The European Union implements classified control on E-cigarettes. The Tobacco Products Directive (TPD) stipulates that E-cigarettes containing nicotine will be included in the control range from May 2016. There are also differences between countries. In Korea, Togo and other countries, E-cigarettes containing nicotine are regarded as tobacco, while in Lithuania, Malta, Thailand, Vietnam and other countries, E-cigarettes are generally regarded as tobacco. 

#### 2.2.2. Pharmaceutical Products

Japan and other countries regard E-cigarettes as pharmaceutical products (see in Table 1). Among them, some countries and regions regard E-cigarettes as medical products, such as Denmark, Estonia, Greece, Hungary, New Zealand, the Philippines, Slovakia, Sweden, and Taiwan, China. Other countries, such as Austria, Canada, Finland, Japan, and Switzerland, consider E-cigarettes to be pharmaceutical products based on whether the e-liquid contains nicotine or tobacco extracts. They regard E-cigarettes containing nicotine to be pharmaceutical products. For example, in France, when the E-cigarette and e-liquid are used for the purpose of quitting smoking, no matter how high the content and concentration of nicotine is, they will be regarded as pharmaceutical products [11]. 

#### 2.2.3. Consumer Products

Italy, Russia, Spain and other countries regard E-cigarettes as electronic products. Switzerland regards E-cigarettes containing no nicotine as ordinary consumer goods. In France, when the E-cigarette and e-liquid are not regarded as pharmaceutical products, E-cigarettes are regarded as ordinary consumer goods, and are regulated by the general consumer product safety act.

#### 2.2.4. Other

Under certain conditions, E-cigarettes may have attributes other than those of tobacco products, pharmaceutical products or ordinary consumer goods. For example, in Australia, Canada, and Malaysia, some E-cigarettes are regarded as being drugs, because the e-liquid contains a certain amount of nicotine. 

### 2.3. Production Control

**Figure 2:**
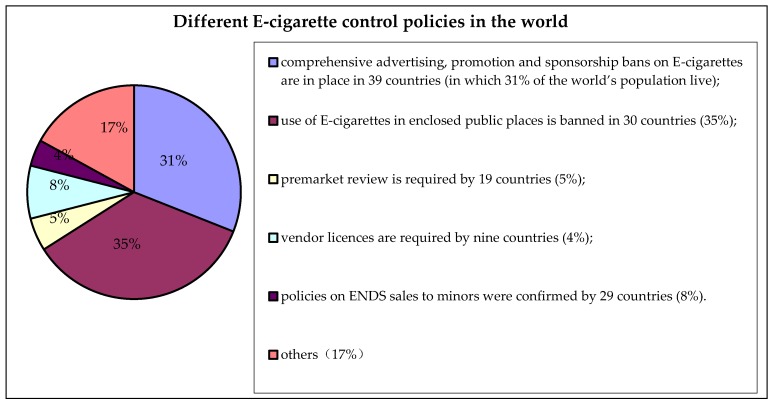
Different E-cigarette control policies in the world [2].

The output of E-cigarettes is huge. The policies and intensity of control are quite different due to the variation in their classification among different countries. At present, there are four main types of policy for the control of E-cigarettes. The first one is prohibition, which means prohibition of the import, sale, usage, advertisement, and public consumption, as well as the sale of E-cigarettes to minors. The second type of policy is to control E-cigarettes as drugs or medical products. The third one is to control E-cigarettes as tobacco products. The final category of policy is to regulate E-cigarettes as electronic products and ordinary consumer goods [12]. The distribution of different E-cigarette control policies in the world are shown in Figure 2 [2].

There are very few countries and regions that regulate the production of E-cigarettes. Brazil, Greece, Israel, the United Arab Emirates, and some local governments in India have banned E-cigarette production. Chile and Malaysia have also introduced management regulations on E-cigarette production. The control of E-cigarettes in China does not exist, and the product quality has no standards, meaning that the E-cigarette industry is in chaos. The quality of E-cigarette products is uneven and without security guarantees, and the hidden dangers behind this situation are worrying [9,13,14]. In a report on the adverse effects of E-cigarette smoke issued by the United States Food and Drug Administration (FDA), it was pointed out that about half of the tobacco-related adverse events reported in more than 100 tobacco reports were related to E-cigarettes [15]. 

In addition, the explosion of electronic smoke has been reported. In 16 January 2017, a man in Pocatello, Idaho State of the United States, lost 7 teeth and got second-degree burns on the left side of his left face due to the sudden explosion of an E-cigarette, and the great power even blew up the wash basin in the bathroom [16]. Thus, although E-cigarettes have only been on the market for a short time, there are many quality and safety problems.

### 2.4. The Harm of E-Cigarettes

#### 2.4.1. Main Components and Hazards of Gas Emissions from E-Cigarettes

The definite components in the e-liquid include propylene glycol, glycerol, nicotine (either included or not), and essence. Han, S.L. et al. determined the contents of the main components in 51 kinds of E-cigarette e-liquid. It was found that 1,2-propylene glycol and glycerol were the main solvents in most of E-cigarette e-liquids, with the content ranging between 75% and 95% [17]. Although 1,2-propylene glycol and glycerol are widely used in the food, pharmaceutical and cosmetic industries, they are also food additives approved by the European Union [18]. Although they are considered safe for oral use, propylene glycol will be converted into propylene oxide during aerosol inhalation, and propylene oxide is classified as a Class 2B carcinogen by the International Agency for Cancer Research. In addition, the glycerol will be converted to acrolein, which causes stimulation of the upper respiratory tract [19]. A short-term exposure study showed that 5 min of E-cigarette use resulted in a significant increase in airway resistance [20]. Another study found that with exposure to E-cigarette smoke, the number of free radicals in the lungs would increase significantly, indicating that E-cigarettes caused the development of oxidative stress in the lung. The content of 8-hydroxy-2-deoxyguanosine in the lungs also increased significantly. The 8-hydroxy-2-deoxyguanosine is widely used as a biomarker to assess oxidative stress and carcinogenicity [21]. Goniewicz, M.L. et al. found four kinds of toxic substances, including carbonyl compounds, volatile organic compounds (VOCs), tobacco-specific nitrosamines (TSNAS), and heavy metals in the analysis of E-cigarette smoke [22]. In addition, the essence, nicotine, and aerosol particles generated by electronic devices are harmful to the health [23].

(1) For the carbonyl compounds, the heating of glycerol will produce harmful aldehydes related to temperature, including formaldehyde, acetaldehyde and acrolein. These three compounds were identified in almost all of the examined E-cigarettes [22]. The study of Gassee, F.R. et al. showed that the mixture of the above three compounds produced more sensory stimuli than those of a single compound [24]. Among them, formaldehyde was categorized as a Class 1 carcinogen in 2006. Acrolein stimulates the nasal cavity, damages the lungs and the inner walls of the blood vessels, and is a major factor leading to cardiovascular disease. Chronic inhalation of acrolein inhibits circulation of endothelial progenitor cells and promotes atherosclerosis, which accelerates the rate of hardening of the aorta by 1.6 times [25]. Ambrus, J.L. et al. also confirmed the presence of acrolein, toxic substances and mutagenic compounds in E-cigarette smoke. It was found that the carcinogenic metabolic enzymes in rats exposed to E-cigarette smoke increased significantly, inducing a greater carcinogenic risk of carcinogens in E-cigarette smoke [26].
(2) Nicotine is the addictive component of tobacco. It induces adverse effects during pregnancy and contributes to the progress of cardiovascular disease. Although nicotine itself is not a carcinogen, it functions as a “tumor promoter”. Nicotine is involved in neurodegeneration and other malignant diseases. Therefore, the use of nicotine in children, adolescents, pregnant women and women of reproductive age should be treated with great caution, due to the effects of nicotine on the brain [2]. The effects of nicotine on the cardiovascular system will be elaborated in the following chapters.
(3) Volatile organic compounds (VOCs) include toluene and meta xylene. In the study of Czogala, J. et al. almost all of the detected smokes contained toluene [27]. In the study of Goniewicz, M.L. et al. toluene and meta xylene were detected in almost all sample smokes. These VOCs are irritating to the skin and mucous membrane, and have an anesthetic effects on the central nervous system, as well as having certain carcinogenicity [22].
(4) *N*′-nitrosonornicotine (NNN), 4-(methylonitrosoamino)-1-(3-pirydyl)-l-butanone (NNK) and *N*′-nitrosoanatabine (NAT) are the three most representative tobacco-specific nitrosamines (TSNAS) in E-cigarettes. Kim, H.J. et al. tested the content of TSNAS in e-liquid samples. There were various kinds of TSNAS tested in 105 samples, among which the maximum concentration of NAT reached 62.19 ug/L, significantly exceeding the requirement for cigarette companies [28]. Nitrosamines have strong carcinogenicity. Moreover, both nitrosamine and the polycyclic aromatic hydrocarbon benzopyrene can interact with DNA. These produced additives interfere DNA replication and the duplicated DNA produces purine free sites and triggers gene mutations [29]. The chemical mixture in the smoke of the E-cigarette will cause chromosome division, and may cause damage to mitotic spindles or filaments, thereby inducing mutations [21].
(5) Heavy metals include lead, nickel, cadmium, and so on. In 2013, Williams, M. et al. found heavy metals such as tin, nickel, lead, chromium and other nanoparticles in the smoke of E-cigarettes [30]. The inhaled heavy metal nanoparticles can be deposited in the alveoli, inducing lung damage and leading to cough, dyspnea, chest pain, pulmonary edema, acute respiratory failure, as well as carcinogenicity, nephrotoxicity, and neurotoxicity [22]. For example, tin is cytotoxic to human lung fibroblasts [30]. In 2017, Williams, M. et al. continued to study the electronic flue gas sol. They selected 36 kinds of elements to detect, of which 35 kinds were detected in the electronic flue gas sol, while there were only 15 kinds of heavy metals detected in traditional cigarettes. These elements contain a variety of heavy metals, and the concentration is usually higher than that of traditional cigarette smoke [31]. The content of lead and chromium is equal to that of traditional cigarette smoke. Especially the nickel content is much higher than that of traditional cigarettes [30]. However, we still do not know the effects of inhaling heavy metal particles in aerosol on health.
(6) There are studies that have investigated the cytotoxicity of the essence in E-cigarettes. Bahl, V. et al. used MTT (3-(4,5-dimethyl-2-thiazolyl)-2,5-diphenyl-2-H-tetrazolium bromide) assays to test the cytotoxicity of e-liquids. The results showed that some e-liquids were cytotoxic to human embryonic stem cells and mouse neural stem cells, and that the composition and concentration of chemical components played an important role. In addition, more evidences confirmed that the chemical properties and the concentration of essence added to the e-liquid, rather than the nicotine alone, are much more harmful [32]. Farsalinos, K.E. et al. used MTT tests to study the in vitro toxicity of the e-liquid of 20 E-cigarettes. The results showed that some electronic smoke liquid had a toxic effect on cardiac muscle cells after heating and atomizing, which would cause disease. The evidence indicated that the ingredients involved in the progress of diseases were related to the production process adopted by the liquid tobacco production company, as well as the flavor components added to cater to the public’s preference [33]. Cinnamylaldehyde and diacetyl are approved flavorings in food, but they will affect people’s health when inhaled [19]. Zeng, W.L. et al. collected tobacco smoke by cell culture medium, and studied the effect of smoke on the relative proliferation rate of Chinese hamster ovary cells (CHO cells) by MTT. The results showed that the relative proliferation rate of flue gas trapping liquid on CHO cells was 10 times higher than that of a 3R4F-reference-cigarette at 100% smoke concentration [34].

#### 2.4.2. The Harm of Second-Hand Smoke from E-Cigarettes

Secondhand smoke produced by traditional cigarettes has many effects on human health, including increased risk of respiratory tract diseases, lung cancer, infectious diseases, acute cardiovascular diseases, and cerebral apoplexy [35]. The secondhand smoke of traditional cigarettes is mainly the side-stream smoke of the cigarette; however, the E-cigarette does not produce side-stream smoke. As a result, the second-hand smoke of the E-cigarette is composed of the exhaled fog of the E-cigarette user. We have evaluated the safety of secondhand smoke produced by E-cigarettes. Schripp, T. et al. placed subjects in a closed chamber to smoke E-cigarettes and exposed them to second-hand smoke. It has been shown that aerosol particles in the electronic smoke enter the lung tissue from the respiratory tract, cycle in the lungs and become smaller particles after being exhaled. In indoor environments, people can also “inhale the steam passively”. The analysis of air components showed that formaldehyde, acrolein, isoprene, acetaldehyde and acetic acid were present, but the chemical compounds produced by traditional cigarette burning were 5 to 40 times than those of the electronic ones [36]. The study results of Goniewicz, M.L. et al. found that the release of smoke from E-cigarettes can increase the amount of nicotine deposition on windows, walls, floors, wood and metal surfaces. Therefore, they believe that smoking E-cigarettes may expose humans to the risk of nicotine indirectly, and future studies should explore the potential risks of carcinogens formed by the nicotine that is released from E-cigarettes [37]. Schober, W. et al. conducted indoor research 6 times. It was found that after 2 h suction of E-cigarettes, the content of PM_2.5_ (particles less than 2.5 microns), propylene glycol, glycerol and nicotine in the indoor air increased significantly. In addition, the total polycyclic aromatic hydrocarbon (PAHs) content, which may cause cancer, increased by 20%, and the concentration of aluminum in the environment increased by 2.4 times [38]. The results of the 5 experiments conducted by Czogala, J. et al. showed that after the use of E-cigarettes, the level of nicotine in the environment was 10% (3.3 compared to 31.6 g/m^3^) that of traditional cigarettes, and the PM_2.5_ concentration was about 18% of that of traditional cigarettes [27]. In the study of Flouris, A.D. et al. and colleagues, 15 non-smokers were exposed to secondhand smoke from traditional cigarette or smoke from E-cigarettes for an hour, and then the cotinine levels in their serum were tested. The results showed that the cotinine levels in the serum of non-smokers were similar after inhaling the secondhand smoke of traditional cigarettes and E-cigarettes (2.6 to 2.4 ng/mL) [39]. As a result, nicotine levels, along with levels of some potentially toxic substances in the body, will increase when they are exposed to the secondhand smoke of E-cigarettes. Although current studies have shown that the risk of secondhand smoke generated by E-cigarettes is expected to be lower than that of traditional cigarettes, there is a high possibility that non-smokers will be exposed to certain doses of nicotine and other toxic substances. With more and more E-cigarettes used in public spaces, a greater diffusion of these toxic and harmful substances will be released into the environment.

#### 2.4.3. E-Cigarettes and Smoking Cessation

Smoking cessation is the most effective way to prevent cardiovascular disease, so it is strongly recommended by guidelines for the prevention and treatment of cardiovascular disease [40]. However, nicotine addiction makes it difficult for most people to quit smoking [41]. Therefore, the current methodology of quitting smoking is a combination of nicotine replacement with the drugs that include nicotinic receptor agonists and behavioral therapy. In conjunction with this treatment, electronic smoke inhalation of nicotine simulates the effect of traditional cigarette smoking. As a result, more and more consumers prefer E-cigarettes when they quit smoking. It is undeniable that E-cigarettes rather than traditional tobacco are much more effective for some people to achieve smoking cessation. However, some people will become a “double user”, namely they smoke both traditional cigarettes and E-cigarettes, especially in places where smoking traditional cigarettes is prohibited. This does not help to quit smoking, but rather leads to greater harm from smoking. Because of the “harmless” nature of E-cigarettes, tobacco users may reuse tobacco products. E-cigarettes may also be a potential way for adolescents or non-smokers to use tobacco, and the use of E-cigarettes may also be the gateway to other drugs or harmful substances. Moreover, users of E-cigarettes are less likely to quit smoking than those who have never used it [19]. As early as 19 September 2008, the WHO declared that E-cigarettes were not proven to be effective for nicotine replacement therapies, and there was no scientific evidence that the product was safe and effective [12]. Most importantly, nicotine is a highly addictive substance. If it cannot be strictly and carefully controlled, the function of the E-cigarette as a tool for nicotine replacement for the purposes of smoking cessation will not be fully achieved [42]. E-cigarettes are packaged as harmless smoking-cessation products by sellers with the suggestion that the amount of nicotine in the e-liquid be gradually reduced, which would help smokers get rid of their nicotine dependence and achieve the goal of quitting smoking [4]. However, the nicotine content of some E-cigarettes is not consistent with the labeling [43]. In the process of smoking E-cigarettes, few people are able to get standardized guidance. Generally, the content of nicotine in the e-liquid is added quantitatively. In order to increase the number of users, different manufacturers have added various flavors of spices, many of which have not been approved by the authorities. Therefore, the efficacy of E-cigarettes for smoking cessation requires further research and demonstration.

### 2.5. E-Cigarettes and Cardiovascular Atherosclerotic Diseases

Smoking is a major independent risk factor for cardiovascular diseases (CVD), and the amount of smoking is an independent risk factor for mortality in patients with coronary atherosclerosis. The total amount of cigarette smoking is significantly associated with coronary artery disease and coronary artery stenosis. There is also evidence showing that either active or passive smoking can constitute a risk factor for cardiovascular disease. Passive smoking increases the risk of CVD by 70% to 80%, and this result is similar to active smoking. The relationship between smoking and cardiovascular disease risk has been confirmed, but the exact mechanism is not clear. The mechanism of atherosclerosis induced by smoking may be related to endothelial cell injury and elevated levels of nitric oxide in the blood. Inflammatory response plays an important role in the occurrence and development of atherosclerosis induced by smoking. It was found that the number of inflammatory cells (such as neutrophils and monocytes) increased significantly in the blood of smokers [44]. Nicotine, as the main harmful component in tobacco, plays an important role in the occurrence and development of atherosclerosis [45]. Although the e-liquid has removed tar and other harmful ingredients, more work needs to be done to evaluate its safety due to the existence of nicotine and other potential hazards such as aerosol particles and different flavor additives in e-liquid.

(1) The harm of nicotine: smoking has become an independent risk factor for the formation of atherosclerosis, and the addictiveness of nicotine is known to all. Nicotine induces the release of catecholamine and cortisol, and causes hemodynamic changes (increase in heart rate, rise in blood pressure, and the vasoconstriction) and adverse effects on blood lipids (leading to activation of adenylate cyclase in adipose tissue and decomposition of triglycerides. The study found that the total lipid composition of the rats exposed to E-cigarettes smoke increased significantly, the content of saturated fatty acids increased significantly, while the content of unsaturated fatty acids decreased significantly), as well as the induction of insulin resistance [19,21]. Nicotine can also cause endothelial dysfunction, inhibit apoptosis, and enhance angiogenesis. This effect raises concerns with regard to nicotine promoting cancer development and accelerating atherosclerosis [46]. Researchers at Danderyd Hospital in Sweden have found that inhaling E-cigarettes only 10 times can cause signs of damage to the blood vessels. Subsequently, they further studied the effects on healthy people of inhaling E-cigarettes 30 times. Magnus Rudbeck, a doctor at Danderyd Hospital who participated in the study, believes that E-cigarette users have poor vascular elasticity, and the poor vascular elasticity may lead to heart disease and stroke. Researchers believe that the nicotine in E-cigarettes may lead to poor vascular elasticity [47]. The study of Flouris, A.D. et al. found that white blood cell count increased after smoking E-cigarettes, which reflected the inflammatory process of acute cardiovascular events [48]. The health status of E-cigarette users was analyzed. It was found that, compared with non-smokers, the former had an enhancement in cardiac sympathetic nerve excitability, and they were more prone to oxidative stress reactions. Professor Choupo Perk from European Society of Cardiology explained that the stimulation of atomized nicotine on sympathetic nerves can cause irregular beating of the heart and elevated blood pressure, and may cause long-term harmful effects on the growth of the vascular wall [49]. Vlachopoulos, C. et al. investigated cardiovascular risk factors among 24 young smokers in 4 different smoking scenarios, and used femoral artery to femoral pulse wave velocity (PWV) to assess aortic stiffness, finding that E-cigarette smoke increased young people’s arterial stiffness and blood pressure, and smoking E-cigarettes for over 30 min had an adverse effect on arterial stiffness that was similar to that of traditional cigarettes [50]. The study of Battista, L. et al. also showed that nicotine inhalation vapors produced the same pathophysiological cardiovascular effects as traditional cigarettes [51]. Farsalinos, K.E. et al. studied the acute effects of E-cigarette smoke on the cardiovascular system of exposed people, and the results showed that the cardiac output blood pressure of people exposed to E-cigarettes (11 mg/mL of nicotine content) increased slightly; when the heart of smokers contracted, cardiac output and heart rate were significantly increased. They also used the MTT test to authenticate that the atomized e-liquid has toxic effect on cultured myocardial cells [33].
(2) The hazards of aerosol particles were also studied. Zhang, Y. et al. showed that 73~80% of the particles were in exhaled aerosols, while 7~18% of the particles were stored in the alveoli. It is estimated that 20~27% of the particles entered the circulatory system, which is equivalent to the proportion for traditional cigarette smoke [52]. Moreover, the size distribution and amount of particles of E-cigarettes are similar to those of traditional cigarettes, and some E-cigarettes produce more particles than traditional cigarettes. Particles may cause lung and systemic inflammation, and increase the risk of cardiovascular disease, respiratory disease and death [5].

## 3. Discussion

Traditional cigarette products have been used for hundreds of years. Although cigarette products have been favored by a considerable number of people, the potential hazards caused by smoking have also been paid a great deal of attention. Under these circumstances, governments have launched a series of policies and regulations to control the use of tobacco. With people paying more and more attention to their health, smoking cessation has been widely considered and accepted by the public. On the other hand, tobacco products are now being developed with greater diversification, and can be smokeless. One important form of new tobacco product, E-cigarette products, have been developed, and have quickly dominated the market share. E-cigarettes don’t burn or produce smoke and don’t contain solid particles or tar, while traditional smoke produces these harmful chemicals. Therefore, E-cigarettes are often considered to be safe, and are allowed to be used in public places in some countries and regions. Since the emergence of E-cigarettes, a number of scientists have done a lot of research to investigate their safety. Nevertheless, there is still no specific conclusion. The variation in the classification and control policies relating to the sale and production of E-cigarettes in different countries makes it more difficult to evaluate their safety. Although E-cigarettes only entered the market a short time ago, their characteristics of being both smokeless and relatively healthy for the environment and for health have led them to be quickly accepted and consumed by the public. At present, the main form of E-cigarette is the renewable liquid type. Due to its being fashionable, economical and practical, this is still the future focus of E-cigarettes in the next decades. Furthermore, different amounts of nicotine are added and used in E-cigarettes in various countries, which counteracts the principles of nicotine replacement therapy advocated by the WHO. Actually, there is no solid evidence showing that smoking cessation could be achieved by using E-cigarettes.

It is only ten years since E-cigarettes were first developed as a commercial product. E-cigarettes are consumed by inhalation through the respiratory tract and air exchange with lung. So it is not clear if E-cigarettes cause damage to the respiratory tract and other parts of the body. Additionally, the small sample sizes in the population survey and the epidemiological statistics analysis have limited the study of their safety. Under the current circumstances, it is of great importance to investigate the influence of E-cigarettes on human health. Furthermore, more information needs to be provided to the public in terms of guidance for the safe and reasonable use of E-cigarettes. For example, whether or not addiction will be induced after long-term use of E-cigarettes is a question that should be paid more attention. The safety of the equipment is another concern. Meanwhile, specific policies should be authorized by governments to guarantee the quality of production process, the specifications of the E-cigarette, and its safety evaluation. The current review provides relevant information useful for understanding the constitution, history and safety evaluation of E-cigarettes based on the existing literature. 

## 4. Conclusions

The harm of E-cigarettes cannot be underestimated. In vivo and in vitro evidence has confirmed that the components contained are harmful to the respiratory system and the cardiovascular system. Moreover, the levels of harmful components, which include volatile organic compounds, tobacco-specific nitrosamines, and heavy metals, in E-cigarettes are higher than those in traditional cigarettes. At present, the research on E-cigarettes is fragmentary and incomplete, and there has been no systematic review of its safety. Additionally, the lack in quality control and authorized policies for E-cigarettes makes it inconvenient to conduct further research and development. 

## Figures and Tables

**Table 1:** Classification of E-cigarettes in different countries [10].

Product Attributes	Countries and Regions
consumer product	Slovenia, Bulgaria, Czech Republic, Ireland, Italy, India, Latvia, Holland, Poland, Russia, Cyprus, Spain, Ukraine, etc.
tobacco product	The United States, Brunei, South Korea, Lithuania, Malta, Singapore, Thailand, Togo, Vietnam, etc.
pharmaceutical product	Austria, Belgium, Canada, Denmark, Luxembourg, Britain, Japan, Finland, France, Greece, Hungary, New Zealand, Taiwan, Estonia, Portugal, Slovakia, Switzerland, Sweden, etc.
